# Characterising Foot-and-Mouth Disease Virus in Clinical Samples Using Nanopore Sequencing

**DOI:** 10.3389/fvets.2021.656256

**Published:** 2021-05-17

**Authors:** Emma Brown, Graham Freimanis, Andrew E. Shaw, Daniel L. Horton, Simon Gubbins, David King

**Affiliations:** ^1^yaDepartment of Transmission Biology, The Pirbright Institute, Woking, United Kingdom; ^2^Faculty of Health and Medical Science, School of Veterinary Medicine, University of Surrey, Guildford, United Kingdom; ^3^Department of Bioinformatics, Sequencing & Proteomics, The Pirbright Institute, Woking, United Kingdom; ^4^Vesicular Disease Reference Laboratory, The Pirbright Institute, Woking, United Kingdom; ^5^Department of Microbial and Cellular Sciences, School of Biosciences and Medicine, Faculty of Health and Medical Sciences, Stag Hill campus, University of Surrey, Guildford, United Kingdom

**Keywords:** foot-and-mouth disease, MinION, nanopore sequencing, characterisation, consensus accuracy

## Abstract

The sequencing of viral genomes provides important data for the prevention and control of foot-and-mouth disease (FMD) outbreaks. Sequence data can be used for strain identification, outbreak tracing, and aiding the selection of the most appropriate vaccine for the circulating strains. At present, sequencing of FMD virus (FMDV) relies upon the time-consuming transport of samples to well-resourced laboratories. The Oxford Nanopore Technologies' MinION portable sequencer has the potential to allow sequencing in remote, decentralised laboratories closer to the outbreak location. In this study, we investigated the utility of the MinION to generate sequence data of sufficient quantity and quality for the characterisation of FMDV serotypes O, A, Asia 1. Prior to sequencing, a universal two-step RT-PCR was used to amplify parts of the 5′UTR, as well as the leader, capsid and parts of the 2A encoding regions of FMDV RNA extracted from three sample matrices: cell culture supernatant, tongue epithelial suspension and oral swabs. The resulting consensus sequences were compared with reference sequences generated on the Illumina MiSeq platform. Consensus sequences with an accuracy of 100% were achieved within 10 and 30 min from the start of the sequencing run when using RNA extracted from cell culture supernatants and tongue epithelial suspensions, respectively. In contrast, sequencing from swabs required up to 2.5 h. Together these results demonstrated that the MinION sequencer can be used to accurately and rapidly characterise serotypes A, O, and Asia 1 of FMDV using amplicons amplified from a variety of different sample matrices.

## Introduction

Foot-and-mouth disease virus (FMDV) is a highly infectious, positive sense single-stranded RNA virus belonging to the family *Picornaviridae* and is the causative agent of foot-and-mouth disease (FMD) (1). FMD remains a globally important livestock disease affecting domestic and wild cloven-hoofed animals (2). FMD remains endemic in parts of Africa, Asia, the Middle East and South America, and periodically causes outbreaks in previously free countries and regions (3). FMD control programs are expensive and trade restrictions prevent the export of animals or animal products from FMD endemic countries. It is estimated that FMD costs between US$6.5–21 billion per year in endemic regions (4). Outbreaks of FMD in free countries can also have a huge economic impact, for example the 2001 UK epidemic has been estimated to have cost the UK economy in excess of £8 billion as a result of agricultural losses (5).

FMDV exists as seven immunologically distinct serotypes; O, A, C, Asia 1, SAT (Southern African Territory) 1, 2, and 3, each with multiple topotypes. Within each topotype there is also a wide spectrum of antigenically distinct intra-serotypic strains, reflecting significant genetic variability (6). Molecular sequence data can provide insights into the genetic relationships between different strains of FMDV and can be used to reconstruct viral transmission pathways (7). Historically, FMDV sequencing has focused upon the VP1 coding region in the FMDV genome, which exhibits ~30–50% variability between the seven serotypes (6). VP1 sequencing can be used to map the geographical distribution of these viruses and monitor transboundary movements of the viruses to identify newly emerging strains (6). Sequencing the capsid coding region of the FMDV genome (which encodes all of the structural proteins; VP1, VP2, VP3, and VP4) allows outbreak tracing to be performed with higher resolution, as well as providing information about antigenic sites that may be missed with VP1 sequencing alone, as a number of the antigenic determinants are located on VP2 and VP3 (8, 9). Increased genetic resolution can also allow the reconstruction of outbreak transmission events on a finer scale (7, 10, 11).

The sequencing platforms routinely used for characterisation of FMDV are limited to use in well-equipped laboratories or core facilities as they require sample preparation, expensive and sophisticated equipment, and computational resources for data analysis (12). Although these platforms can provide an accurate and in-depth analysis of sample sequences, samples must be transported to the laboratory, potentially negatively affecting the quality of samples and may delay important decision making with regards to disease control (13). This is particularly the case for FMDV where in many endemic settings resources are often limited and there can be considerable logistical challenges in getting samples to a laboratory in a timely manner. Yet cheap and rapid generation of sequence data could help countries within the FMD progressive control pathway to improve the monitoring and control of outbreaks, for example, through identification of circulating strains and the use of better matched vaccines. In 2014, Oxford Nanopore Technologies (ONT) released the MinION, a small portable sequencing device which uses a single-molecule sequencing approach to produce long sequence reads from input nucleic acid. The sequencer is powered via a computer USB port and is not reliant upon an internet connexion (14). In addition to its size, the MinION device is a low cost alternative to other current sequencing options such as those offered by Illumina (15). The MinION has been used to detect a variety of viral pathogens, for example in Guinea and Liberia during the Ebola outbreak in West Africa in 2015 (16, 17) and more recently during the ongoing COVID-19 pandemic (18). The MinION has formed a part of mobile laboratories with a workflow that included amplification, sequence library preparation and sequencing of samples which could be carried out within 2 days (16–18). The possible use of the MinION in field settings was also explored during the Zika outbreak in South America in 2016 (19, 20).

To date, the characterisation of FMDV in remote locations has been limited to serotype or lineage-specific RT-PCR assays. Although sensitive and specific, the technology is limited to a pool of viruses circulating in a defined region, and as such may not be able to identify new emerging strains of FMDV (21, 22). In addition, typing PCRs do not provide sequence data suitable for studying the evolution of the genome or vaccine matching (23). In this study, we have investigated the efficiency of the MinION to generate sufficient sequence data for characterisation of FMDV in different sample matrices.

## Materials and Methods

### Sample Selection

FMDV strains representative of serotypes O (O/UKG/34/2001), A (A/TAI/17/2016), and Asia 1 (ASIA1/IRN/49/2011) were selected for this study. O/UKG/34/2001 (henceforth “O/UKG”) was first isolated from an abattoir during the UK epidemic in 2001 (24). A/TAI/17/2016 (henceforth “A/TAI”) was isolated from an outbreak in Thailand in 2016 and ASIA1/IRN/49/2011 (henceforth “ASIA1/IRN”) is derived from an outbreak in Iran in 2011.

Three sample matrices were explored for their suitability: cell culture supernatant, tongue epithelium, and oral swabs. All cell culture adapted viruses were passaged on porcine renal (IB-RS-2) or bovine thyroid (BTY) cell lines maintained at The Pirbright Institute and kept as archival stocks (Supplementary Table 1). The clinical samples (tongue epithelium and oral swabs) were archival samples from previous cattle experiments (25) carried out at The Pirbright Institute (Supplementary Table 2).

### Tissue Sample Processing

The raw epithelial tissue samples were processed into suspensions for use in the study. The tissue from each animal was weighed (0.25 and 1.17 g from A/TAI-EPI-1 and A/TAI-EPI-2, respectively) and processed in parallel in separate class II microbiological safety cabinets (MBSC). Each tissue was homogenised to a paste using a pestle and mortar with 800 μl of Dulbecco's Modified Eagle's Medium (DMEM) (Sigma Aldrich, UK) and 2 g of sterile sand (Sigma Aldrich, UK). A 10% (w/v) tissue suspension was prepared by suspending the tissue homogenate in a suspension solution [1:1 ratio of M25 phosphate buffer (in house) and glycerol (Sigma Aldrich, UK) supplemented with 10% phenol red (Sigma Aldrich, UK)]. Tissue homogenates were clarified by centrifugation at 3,000 rpm for 10 min at 4°C prior to aliquoting and storage at −80°C.

### RNA Extraction

Viral RNA was extracted from samples using the RNeasy Mini Kit (Qiagen, UK), as per the manufacturer's instructions. High titre cell culture adapted viruses were extracted on a separate day to the epithelium and oral swabs and each strain was extracted separately to avoid cross contamination of the serotypes.

### Molecular Amplification of the P1 Coding Region

Firstly the samples were screened by RT-qPCR to confirm the presence of FMD viral RNA. Secondly, a PCR amplification approach was used to generate the required input, 1 μg dsDNA, as stated by the MinION sequencing protocol (26). A universal two step protocol to amplify the P1 coding region of FMDV was used as previously described (9).

#### Reverse Transcription

Reverse transcription (RT) of the RNA was performed using SuperScript™ IV First-Strand Synthesis System (Invitrogen, UK), as per the manufacturer's instructions, with the addition of 20 μl of a gene specific primer; Rev 6 (GGC GGC CGC TTT TTT TTT TTT TTT) at a concentration of 2 μM (Sigma Aldrich, UK), as previously described (7). RNA isolated from each cell culture supernatants were reverse transcribed separately to the epithelium and oral swabs to avoid cross contamination of the samples.

#### PCR

PCR reactions were assembled using the primers previously described by Xu et al. (9). Each reaction comprised 25 μl of 2X Platinum SuperFi PCR Master Mix (Invitrogen, UK), 17 μl of nuclease-free water (Ambion, Invitrogen, UK), 3 μl of the cDNA template and 2.5 μl of each universal FMDV primer at a final concentration of 0.5 μM (Univ F: TGGTGACAGGCTAAGGATG and Univ R: GCCCRGGGTTGGACTC (Sigma Aldrich, UK). The cycling conditions consisted of an initial denaturation at 98°C for 30 s, followed by 39 cycles of denaturation at 98°C for 15 s, annealing at 66°C for 15 s and extension at 72°C for 30 s, followed by a final extension at 72°C for 5 min.

PCR products were electrophoresed at 80 V for 90 min on a 1% agarose gel stained with 1X GelRed (Thermo Fisher Scientific, UK) in 1 X Tris-acetate EDTA buffer (Thermo Fisher Scientific, UK). The gel was visualised under UV illumination to confirm the presence of a ~3 kb amplicon representing P1.

The PCR products were purified using the QIAquick PCR Purification Kit (Qiagen, UK) as per the manufacturer's instructions and quantified on the Qubit v3 fluorometer using the dsDNA HS (High Sensitivity) Assay Kit (Invitrogen, UK).

### MinION Sequencing

Two sequencing runs were carried out using the MinION sequencer, with the first run containing all cell culture derived material (run one) and the second containing the clinical samples (run two).

On the first sequencing run, libraries were prepared with Oxford Nanopore's PCR barcoding expansion (EXP-PBC001) with the ligation sequencing kit (SQK-LSK109), using the 1D PCR barcoding genomic DNA protocol as per the manufacturer's instructions, with the exception of the Platinum Taq Polymerase which was replaced with Q5 high fidelity DNA polymerase (NEB, UK). The cycling conditions were also adjusted and consisted of an initial denaturation for 30 s at 98°C, followed by 36 cycles of denaturation for 10 s at 98°C, annealing for 30 s at 62°C and extension for 1 min at 72°C, with a final extension step for 2 min at 72°C. The sequencer was run for 30 h with local basecalling using the MinKNOW (version 1.4.22) software.

On the second sequencing run, one epithelium and oral swab sample were selected from each strain of FMDV. The samples selected had differing concentrations of DNA, as determined by the Qubit v3 fluorometer (Supplementary Table 3) to assess the ability of the MinION sequencer to detect samples with a range of DNA concentrations. Libraries were prepared as described for the first sequencing run but with a slight adjustment to the barcoding PCR mastermix. The PCR mastermix was altered to consist of: 2 μl PCR barcode, 2 μl 10 ng/μl adapter ligated DNA, 12.5 μl Q5 high fidelity DNA polymerase and 8.5 μl of nuclease-free water.

### Illumina MiSeq Sequencing

The PCR amplicons from the three cell culture supernatant samples were sequenced on the Illumina MiSeq sequencing platform (regarded in this study as the gold standard) to generate reference sequences. These consensus sequences were visually compared in BioEdit (version 7.2.5) with the MinION derived consensus sequences to determine consensus accuracy. Libraries were prepared using the Nextera XT kit (Illumina, UK) on a NGSSTAR^™^ robot (Hamilton Robotics, UK). Final libraries were multiplexed and diluted to 12.5 pM prior to sequencing on an Illumina MiSeq platform using a single 2 × 150 cycle, paired-end sequencing run using a version 2 chemistry MiSeq reagent cartridge.

### Data Analysis

#### MiSeq Data

Quality control cheques were performed on the MiSeq generated raw reads using the FastQC programme (27) (version 0.11.5). The first 15 bp of the 5′ end and last 5 base pairs (bp) of the 3′ end of each read was removed using Prinseq-lite (version 0.20.4) (28) due to a lower qScore quality. Following this, reads were processed through sickle (Version 1.33) (29), removing reads below 70 bp in length and a qScore below q30 from the data. A reference sequence was assembled using IDBA_UD (30) (version 1.1.1) and the resulting contig confirmed using a BLASTn search. The contig was used as a reference sequence for the alignment of the MiSeq reads using BWA-MEM (version 0.7.12-r1039) (31). The.SAM file produced from the alignment was converted into a.BAM file using SAMtools (32) (version 1.9) from which coverage data and consensus sequences were produced. The authenticity of the resulting consensus sequences was confirmed using a BLASTn search.

#### MinION Data

FASTQ reads were de-multiplexed using the EPI2ME software (version 2019.7.9) from ONT. The reads below a qScore of 7 were removed by the EPI2ME software (as per ONT pass/fail threshold) before onward analysis. Reference genomes for all samples were assembled using Canu (version 2.0) (33) with the default settings (a minimum read length input of 1,000 bp). Following this, Medaka (version 0.11.5) was used to generate a polished consensus sequence. Briefly, Minimap2 (version 2.17) was used to align reads to a reference sequence (generated by Canu) and SAMtools (version 1.9) was used to generate.BAM files from the alignment. Following this, BCFtools (version 1.10.2) was used to generate a consensus sequence from the.BAM file. The consensus sequences were polished using the Medaka model (based on the r9 flowcell). BEDTools (version 2.26.0) (34) was used to ascertain coverage across the sequenced amplicon. The ClustalW Multiple alignment within BioEdit (version 7.2.5) was used to visualise and compare each polished consensus sequence with their respective reference sequence generated on the MiSeq to determine consensus accuracy. The time taken to reach the most accurate consensus sequence is defined as the sequencing time required to generate sufficient reads to assemble a consensus sequence and does not include the bioinformatics analysis time. The.BAM files generated from the alignment were used to determine the proportion of reads under and over 500 bp in length which aligned after 30 h of sequencing.

## Results

### Two-Step PCR for P1 Amplification

The 3D region of the FMDV genome was detected in all samples by RT-qPCR to confirm FMDV viral RNA was present in the samples selected for the study (data not shown).

PCR reactions from the P1 assay were analysed using agarose gel electrophoresis to confirm authenticity of the amplicon. Clear bands of the expected size (~3 kb) were visible for every sample amplified, confirming the successful amplification of the P1 region (data not shown).

The full requirement of DNA needed for MinION sequencing (1 μg) was yielded using template derived from cell culture supernatants and epithelium samples infected with A/TAI and ASIA1/IRN and from one of the two O/UKG epithelium samples (O/UKG-EPI-1). None of the oral swabs (for any serotype) yielded the required 1 μg of DNA. As a result of the limited DNA, MinION libraries were prepared for the oral swab samples using a range of DNA concentrations (Supplementary Table 3).

### Sequencing FMDV Using the MinION

A MinION run of PCR products amplified from cell culture supernatants resulted in a total of 2.16 × 10^6^ reads being generated from 1,227 active sequencing pores. Of these reads, 2.01 × 10^6^ (94%) reads passed the QC philtre. Alignment of the reads against their respective reference sequences revealed that 5.22 × 10^5^ (26%) reads were identified as originating from FMDV O/UKG, 4.19 × 10^5^ (21%) from A/TAI and 4.31 × 10^5^ (21%) from ASIA1/IRN. While 6.42 × 10^5^ (32%) of reads did not align to any of the FMDV reference genomes.

A run using PCR products amplified from clinical material resulted in a greater amount of data being produced (3.55 × 10^6^ reads from 564 active pores), and a higher percentage (99%) of reads passed QC cheques (3.51 × 10^6^). However, “clinical” reads showed a reduced mapping frequency against their respective reference sequences: 2.73 × 10^5^ (8%) reads were identified as originating from O/UKG, 4.50 × 10^5^ (13%) from A/TAI and 8.64 × 10^5^ (25%) from ASIA1/IRN. Similarly, a higher proportion of reads (55%) derived from clinical samples failed to align to the FMDV reference genomes.

#### Alignment Statistics

**Figure 1:**
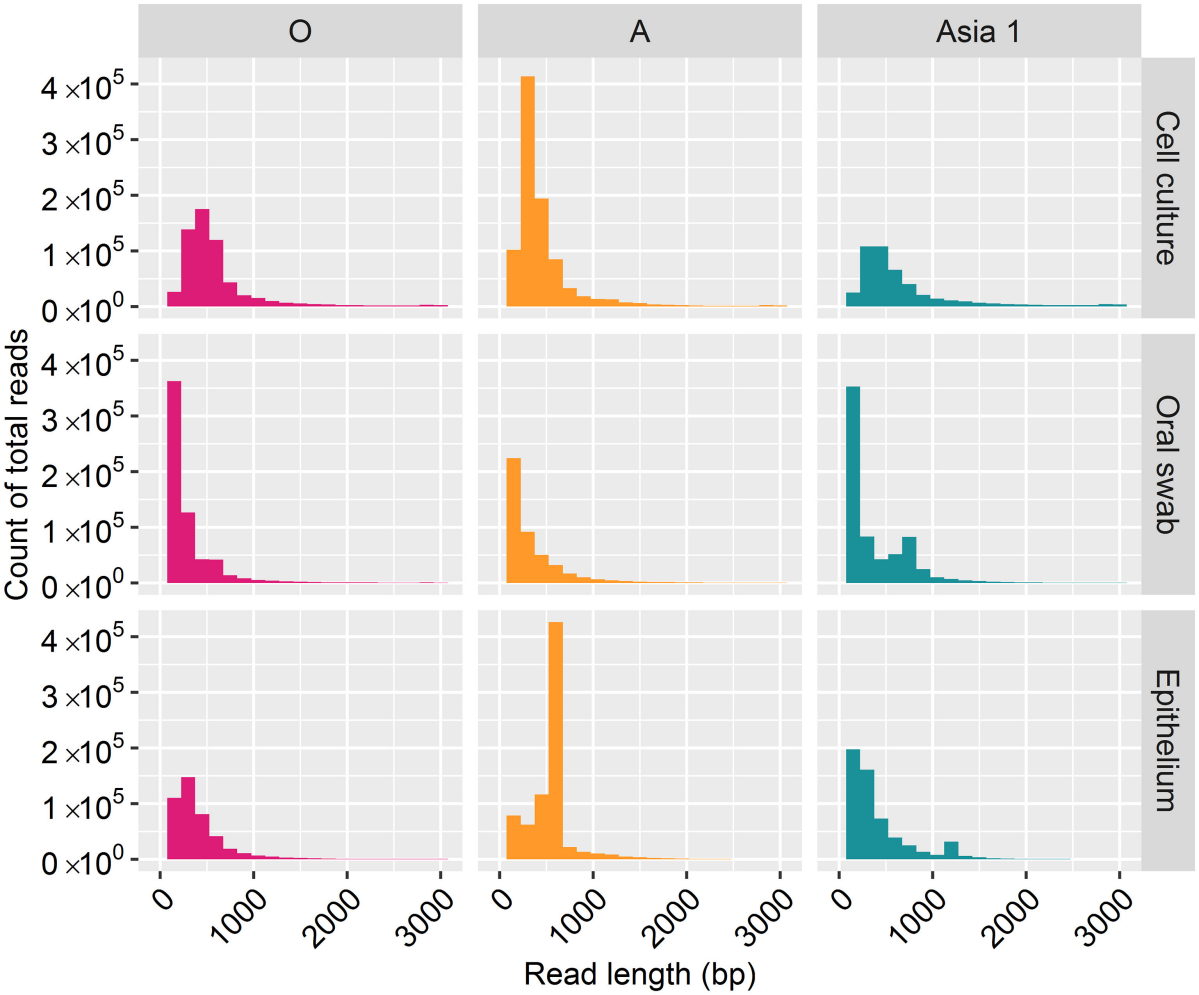
Frequency distribution of MinION read lengths from each sample generated after 30 h of nanopore sequencing.

For samples of cell culture origin, nanopore sequencing resulted in reads ranging in length between one bp and 3,000 bp (Figure 1). However, the majority of reads (70%) were under 500 bp in length, with lengths between 2,800 and 3,000 bp accounting for just 7% of the total reads. In line with the cell culture data, the majority of reads (82%) derived from clinical samples were under 500 bp in length, with reads between 2,800 and 3,000 bp in length accounting for 1% of the total reads.

**Figure 2:**
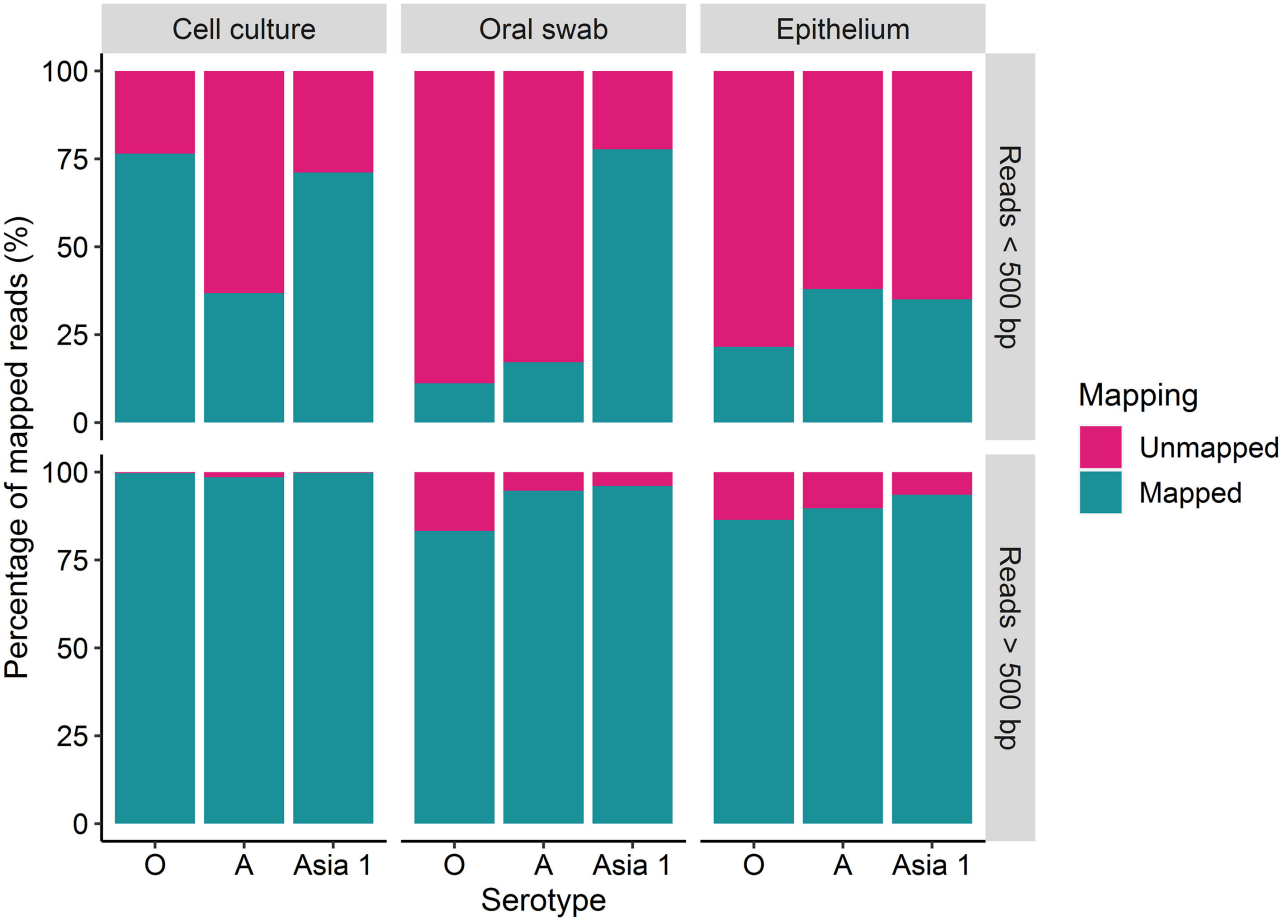
The percentage (%) of MinION reads which were over and under 500 bp in length which mapped to the MinION derived reference sequence using reads generated after 30 h of nanopore sequencing.

The percentage of reads under and over 500 bp that mapped to the reference sequences was variable amongst the different strains (Figure 2). A higher percentage of the longer reads (over 500 bp) mapped to the reference sequences in comparison to the shorter reads (under 500 bp) for each sample tested (Figure 2). Overall, the percentage of mapped reads was higher for cell culture derived samples than for clinical material (Figure 2). Between 83 and 99% of the reads over 500 bp aligned to the reference sequence for FMDV O/UKG, A/TAI and ASIA1/IRN, with a mean of 94% (Figure 2). The percentage of reads under 500 bp which mapped to the reference sequence varied between 11 and 77%, with a mean of 42% (Figure 2).

#### Coverage

**Figure 3:**
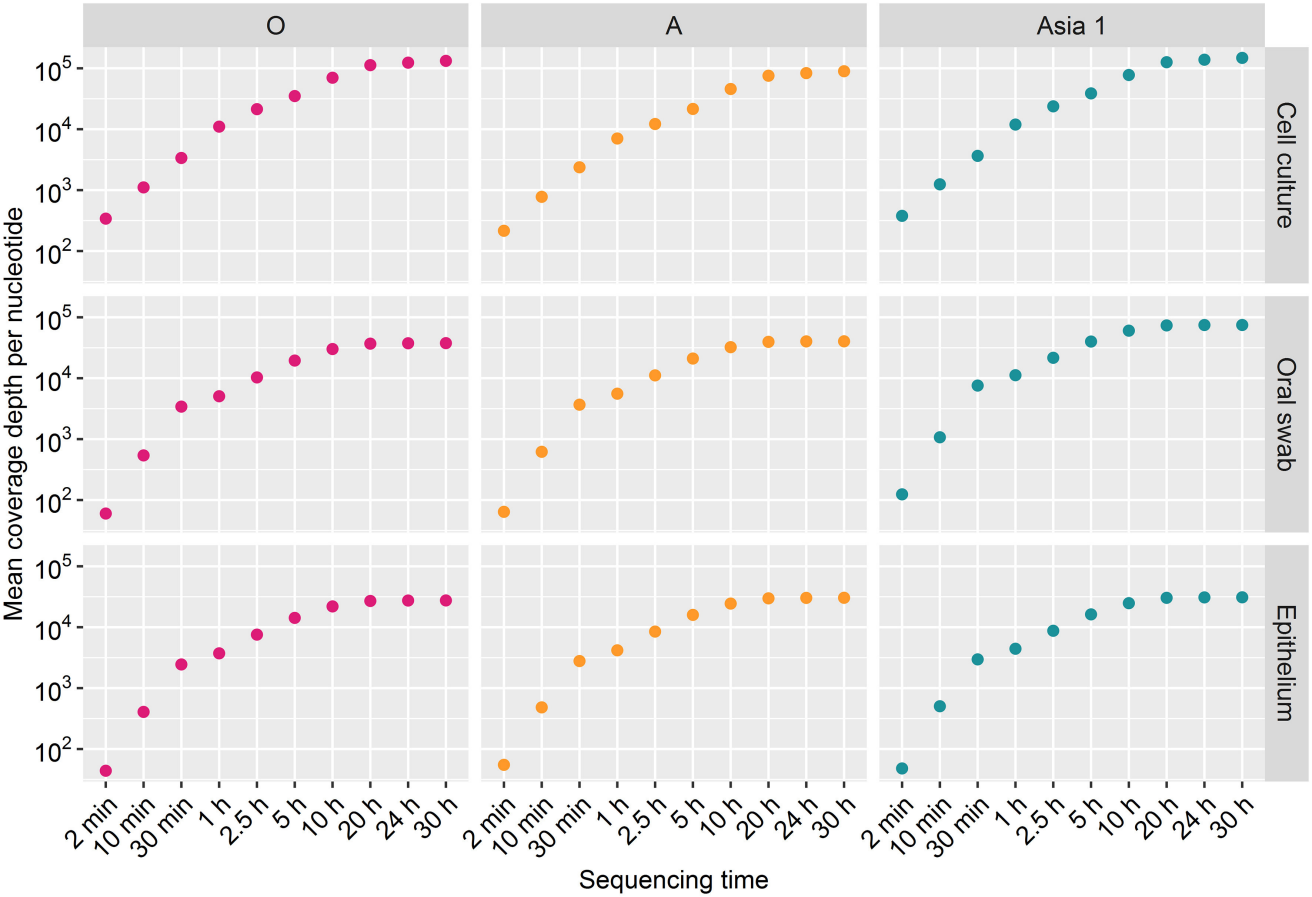
The mean coverage per nucleotide for each sample tested over the duration of the MinION sequencing runs.

Coverage for samples O/UKG-CC and ASIA1/IRN-CC increased at a similar rate, whereas sample A/TAI-CC had a marginally lower coverage throughout the run. A mean coverage depth across the amplicon of 1.32 × 10^5^, 8.95 × 10^4^, and 1.48 × 10^5^ was produced for O/UKG-CC, A/TAI-CC and ASIA1/IRN-CC, respectively, following 30 h of sequencing (Figure 3). In line with the higher mapping rate, the coverage was higher for cell culture derived samples than clinical material. The coverage obtained for oral swabs and epithelium samples across each time point was generally comparable, with the exception of the Asia 1 samples where a higher coverage was achieved for the oral swab than epithelium sample. Overall, the mean coverage for oral swabs and epithelium samples was 5.09 × 10^4^ and 2.96 × 10^4^, respectively, after 30 h of sequencing (Figure 3).

**Figure 4:**
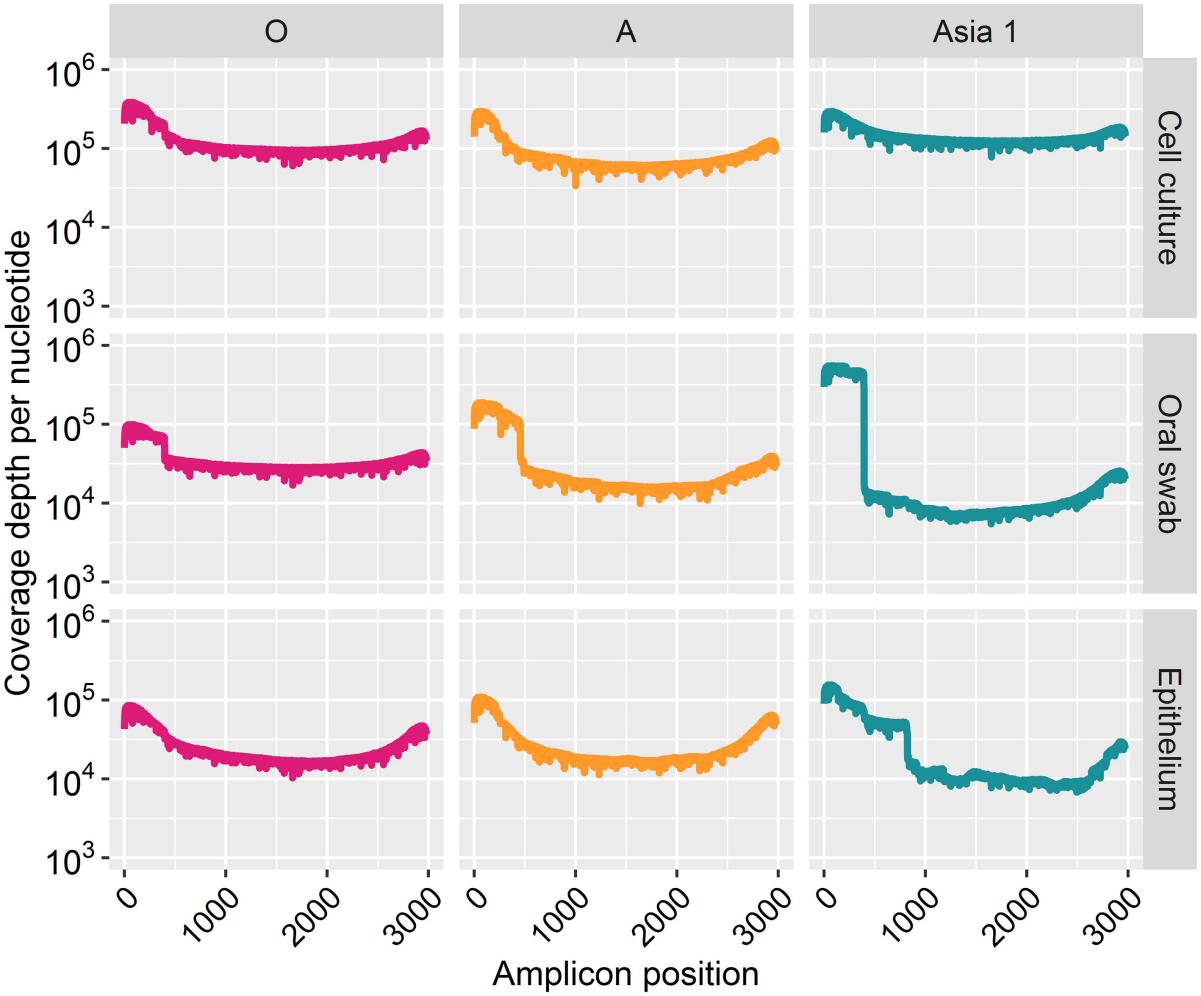
Coverage depth at each nucleotide position of the sequenced amplicon for every sample after 30 h of nanopore sequencing.

Coverage for all samples increased across the sequencing run and plateaued after 20 h of sequencing (Figure 3). The coverage depth was higher for cell culture derived samples than clinical material for each strain (Figure 4). A greater coverage depth was observed at the 3′ and 5′ end of the amplicon for all samples, particularly the clinical samples from cattle infected with ASIA1/IRN. Nevertheless, a whole amplicon sequence was achieved for each sample (Figure 4).

#### Time to Consensus

Reference sequences were generated for each cell culture derived PCR product by assembling a consensus sequence of reads generated using Illumina sequencing on the MiSeq. Consensus accuracy was then defined as how closely the nanopore-based consensus matched the respective MiSeq reference sequence.

Each polished nanopore-based consensus sequence achieved the full amplicon length (2,989, 2,987, 2,985 kb for O/UKG, A/TAI and ASIA1/IRN, respectively) at each time point. All samples achieved a consensus sequence with 100% identity to their respective reference sequences at one time point or more, with the exception of A/TAI-OS and O/UKG-CC where the most accurate consensus was 99.97% achieved after 2 and 10 min of sequencing, respectively, as shown by the point sitting just below the 100% line on Supplementary Figure 1. The sequencing time required to reach a consensus sequence with 100% accuracy varied, with samples A/TAI-CC, ASIA1/IRN-CC, O/UKG-EPI and A/TAI-EPI requiring just 2 min of sequencing. In contrast, O/UKG-OS, ASIA1/IRN-OS and A/TAI-EPI required 2.5 h of sequencing to reach 100% accuracy.

The consensus accuracy fluctuated for all samples over time, the ASIA1/IRN-OS sample proved the most consistent with a continual accuracy of 100% reached after 30 min. The largest fluctuation was observed in O/UKG-EPI with accuracy dropping from 100% after 1 h to 99.80% after 2.5 h, before rising to 99.97% following 5 h of sequencing. The base discrepancies responsible for the drop in consensus accuracy after 2.5 h where identified at homopolymeric regions consisting of sequential G or C bases.

A single discrepancy between the nanopore derived consensus sequences generated beyond 30 min and the MiSeq reference sequence was observed for all samples infected with O/UKG. At amplicon position 75, within the 5′ UTR of the FMDV genome, the MinION sequencer called an A base, whereas the MiSeq called a G base. A single base deletion relative to the MiSeq reference sequence at amplicon position 212, located within the L^pro^ of the FMDV genome, was observed in consensus sequences generated after 5 h and onwards for A/TAI-CC and A/TAI-OS-2, whereby the nanopore derived sequences lacked a T compared to the MiSeq reference sequence. The alternative nucleotide called in the nanopore data resulted in a non-sense change from leucine to a termination codon (TTG to TGA). No discrepancies were observed at the same base position in multiple samples for ASIA1/IRN.

## Discussion

The extensive diversity within and between FMDV serotypes requires the characterisation of many isolates to carry out a detailed epidemiological analysis and as such can complicate the control of the disease (6). Rapid point of care testing and characterisation of pathogens is increasingly being employed to help aid the surveillance and control of infectious diseases, for example FMD, COVID-19, Ebola virus disease and a wide range of arboviruses (16, 18, 21).

The aim of this study was to assess the suitability of the MinION sequencing platform as a tool for FMD characterisation. The results show the MinION was able to generate a consensus sequence with 100% identity to the reference sequence for all bar two of the samples tested. Consensus sequences with 100% identity to the reference sequence after 2 min of sequencing was achieved for almost half (4/9) of the samples tested. The consensus sequences generated after 2 min which did not achieve 100% accuracy were entered into a BLASTn search and in every instance the search revealed the same phylogenetic placement but with a lower percentage identity. Thus, confirming that accurate sequencing data can be generated within a short period of time. Similarly, Quick et al. (16) generated sufficient reads after an hour of sequencing for half of the Ebola cases tested on the MinION, during the Ebola outbreak in Guinea in 2015 allowing the rapid detection of new introductions of the virus from a different geographical location. The findings of this study are important in the context of rapidly characterising FMDV outbreak strains in order to inform the implementation of control measures, a key factor in limiting the economic impacts of FMD (4). Sequence data could also be used to establish transmission chains and identify missing infected premises (7, 35). Importantly, our results can be used to inform the optimum sequencing time required, which could be useful in developing sequence protocols in situations where reference sequences are not available. The results presented here suggest that the sequencing time required to reach the most accurate consensus sequence on the MinION varies depending on sample type with 10 and 30 min of sequencing required when sequencing cell culture supernatants and epithelium, respectively. When sequencing from oral swabs our results suggest this time should be increased to 2.5 h. However, it is difficult to make firm judgements with the number of samples sequenced in this study and a larger follow-on study would be beneficial to strengthen these recommendations. Additionally, our results provide important information regarding the most suitable clinical sample matrix for the sequencing pipeline. In this study, accurate consensus sequences were generated most quickly from epithelium samples, implying that this is the clinical sample-type of choice for rapid sequencing. Nevertheless, our data confirms that it is possible to generate high quality consensus sequences from simple oral swab samples, which provides a larger window for sample collection as samples can be taken before the development and after the healing of lesions from which epithelium is harvested (2).

Despite the high consensus accuracy of the MinION demonstrated in this study single-nucleotide polymorphisms (SNPs) and indels (deleted or inserted bases) were observed between the consensus sequences generated by the MinION and the reference sequences generated on the MiSeq for all samples tested. Specific SNPs or indels were observed in more than one sample from the O/UKG and A/TAI strains at one or more time points, despite using error corrected consensus sequences. The most likely explanation for the observed changes is the widely reported high error rates (5–10%) (36, 37) associated with MinION sequencing as certain examples were non-sense mutations. A deletion of a T base at position 212 in the A/TAI samples resulted in a stop codon, a highly unlikely natural change considering that this position is within the polyprotein region encoded by the FMDV genome (38). All SNPs observed in more than one sample were at the end of homopolymeric regions or GC rich regions, which the MinION is known to be less efficient at sequencing (39). In this study the reference sequences were generated by sequencing the cell culture supernatants on the MiSeq sequencer. This approach does not account for mutations in the viral genome which may have occurred during the replication of the viruses within the host. It is possible that the base changes simply reflect the predominant nucleotide at these positions in an *in vivo* setting, with the exception of the non-sense mutation discussed above.

Complete coverage was observed across the entire amplicon for all samples. The depth of coverage observed was slightly higher for cell culture viruses than clinical samples but followed the same pattern. Interestingly, a higher depth of coverage was observed at the first and last 500 bp of the sequenced amplicon. We expected to observe a high proportion of reads between 2.8 and 3 kb in length (9). However, 70% of reads from cell culture derived template and 82% of reads from clinical material were under 500 bp in length. The consensus sequences for all samples have a GC rich region at positions 494–507 (within the L^pro^ fragment of the FMDV genome). It is possible that GC-rich regions caused the sequencing to terminate after the first 500 bp for the majority of the reads produced. GC-rich regions also coincided with reduced depth in the data generated here with coverage dropping at ~750 bp and a GC rich region identified at positions 748–757 (also within the L^pro^ fragment) for all samples. It has been highlighted by Krishnakumar et al. (40) that one direction reads (1D), such as those produced in this study, are more commonly shorter than one directional squared reads (a strand of DNA is sequenced, followed by the commentary strand). Therefore, a nick on a 1D read would cause the sequencing to halt, resulting in shorter reads (40). Another possibility is the DNA may have become slightly fragmented during the purification of the PCR products prior to sequencing. The use of a magnetic bead instead of a spin column purification method could be used to determine if the purification method influences read length. We have considered why a higher coverage was observed at the last 500 bp. One possible explanation was a G-C rich region or particular motif at the last 500 bp caused sequencing to halt, resulting in reads which entered the pore in the 3′ orientation to be cut short. However, no correlation with G-C regions or an immediate drop in coverage was observed to support this theory. A higher proportion of reads which were over 500 bp in length mapped to the MinION derived reference sequence (with a mean coverage of 94%) in comparison to reads below 500 bp in length (which had a mean coverage of 42%). This could be the result of longer reads containing less error and therefore mapping more efficiently to the MinION derived reference sequence (generated in Canu). It has been suggested that shorter DNA fragments might be more prone to error-inducing stochastic movements during pore translocation such as random fluctuations in a particle's position when suspended in medium, a phenomenon known as the Brownian motion (40). In contrast, longer fragments are less affected, allowing for more consistent movement through the pores.

During outbreaks of FMD the value of a simple positive/negative result in endemic countries is limited and rapid characterisation of circulating strains that can be carried out at local laboratories allows more specific, tailored control strategies to be applied. We envisage that epithelium or oral swab samples could be collected from animals in locations where FMD is suspected or know to be present. Following transport to a local laboratory, a benchtop or portable thermocycler could be used to perform the PCR assay and library preparation needed prior to sequencing on the MinION, as described in this study. The proposed pipeline could be a useful tool in controlling outbreaks of FMD in several scenarios, for example to establish transmission pathways between domestic animals and wildlife species at domestic/wildlife interfaces. Such data could be used to implement restrictions on communal grazing and barriers at these interfaces. Furthermore, rapid and accurate sequence data obtained from the MinION could be used to help make rapid and informed assessments at a local level on the suitability of currently used vaccines in the region. It would also facilitate incorporation of local knowledge when interpreting the sequence data as part of outbreak investigations.

When outbreaks occur in disease-free countries, sequence data would be of most use in constructing transmission trees and, hence, indicating the existence of potentially undetected infected farms (7, 35). As rapidity of testing is critical in this context, local sequencing of samples from affected farms could speed up this analysis by removing the need to send the samples to a central laboratory for analysis.

The present study serves as a proof-of-concept study which could be developed further with a larger sample set, incorporating a greater number of oral swabs and epithelium samples, as well as assessing other sample matrices such as nasal swabs, whole blood, milk and environmental swabs. This pipeline could also be assessed on its suitability with other FMDV serotypes (C, SAT 1, SAT 2, and SAT 3). Whilst the work described here was performed in a laboratory, future research could focus upon modifying the pipeline for use in decentralised locations. The use of the MinION sequencer could be investigated in conjunction with a range of protocols and equipment that have enabled the mobilisation of laboratory procedures and shorten turnaround times, for example a portable PCR platform and the rapid sequencing kit or lyophilised field sequencing kits that are now available from ONT (41). Alternatively the use of lamPORE, which combines RT-LAMP with the rapid sequencing kit and has recently been described for SARS-CoV-2 detection, could be investigated for its suitably for FMD detection and characterisation (42, 43).

The work described here could be expanded upon to develop a sample processing pipeline to generate whole genome sequences of FMDV, for example using tiled primers to amplify the complete genome prior to sequencing. This approach has recently been successful in characterising the SAR-CoV-2 genome (https://artic.network/ncov-2019). Alternative uses of this pipeline in FMD control and surveillance could also be investigated, such as using the FMDV universal PCR followed by MinION sequencing to detect recombination in co-infected populations.

## Data Availability Statement

The datasets presented in this study can be found in online repositories. The names of the repository/repositories and accession number(s) can be found at: https://www.ncbi.nlm.nih.gov/bioproject/693428.

## Author Contributions

EB and DK: conceptualisation. EB, DK, and GF: investigation and formal analysis. EB, DK, and AS: visualisation. EB, DK, DH, GF, AS, and SG: writing—original draught, review, and editing. All authors contributed to the article and approved the submitted version.

## Conflict of Interest

The authors declare that the research was conducted in the absence of any commercial or financial relationships that could be construed as a potential conflict of interest.
