# Supplementary material for: Characterising Foot-and-Mouth Disease Virus in Clinical Samples Using Nanopore Sequencing
Source: Front Vet Sci. 2021 May 17;8:656256. doi: 10.3389/fvets.2021.656256 (PMC8165188; doi:10.3389/fvets.2021.656256)
Supplement: Supplementary file 1 [file Table_1.DOCX]

**Table S.1:** List of cell culture supernatants used in the study

| Virus strain | Sample ID | Details of passage |
| --- | --- | --- |
| O/UKG/34/2001 | O/UKG-CC | Passaged once in bovine thyroid (BTY) cells. |
| A/TAI/17/2016 | A/TAI-CC | Passaged three times in swine renal cells (IB-RS-2) cells. |
| ASIA1/IRN/49/2011 | ASIA1/IRN-CC | Passaged three times in swine renal cells (IB-RS-2) cells. |
