# Supplementary material for: Characterising Foot-and-Mouth Disease Virus in Clinical Samples Using Nanopore Sequencing
Source: Front Vet Sci. 2021 May 17;8:656256. doi: 10.3389/fvets.2021.656256 (PMC8165188; doi:10.3389/fvets.2021.656256)
Supplement: Supplementary file 2 [file Table_2.DOCX]

**Table S.2**: List of clinical samples used in the study.

| Strain | Sample type | Sample ID | Route of challenge | Time of sample collection (days post challenge) |
| --- | --- | --- | --- | --- |
| O/UKG/34/2001 | Oral swab | O/UKG-OS-1 | Needle inoculation | 1 |
|  | Oral swab | O/UKG-OS-2 | Needle inoculation | 2 |
|  | Oral swab | O/UKG-OS-3 | Direct contact | 1 |
|  | Oral swab | O/UKG-OS-4 | Direct contact | 2 |
|  | Tongue epithelium | O/UKG-EPI-1 | Environmental | 6 |
|  | Tongue epithelium | O/UKG-EPI-2 | Environmental | 10 |
| A/TAI/17/2016 | Oral swab | A/TAI-OS-1 | Needle inoculation | 4 |
|  | Tongue epithelium | A/TAI-EPI-1 | Needle inoculation | 5 |
|  | Oral swab | A/TAI-OS-2 | Needle inoculation | 4 |
|  | Tongue epithelium | A/TAI-EPI-2 | Needle inoculation | 6 |
|  | Oral swab | A/TAI-OS-3 | Needle inoculation | 4 |
|  | Oral swab | A/TAI-OS-4 | Needle inoculation | 4 |
| ASIA1/IRN/49/2011 | Oral swab | ASIA1/IRN-OS-1 | Needle inoculation | 2 |
|  | Oral swab | ASIA1/IRN-OS-2 | Needle inoculation | 2 |
|  | Tongue epithelium | ASIA1/IRN-EPI-1 | Needle inoculation | 3 |
|  | Oral swab | ASIA1/IRN-OS-3 | Needle inoculation | 2 |
|  | Oral swab | ASIA1/IRN-OS-4 | Needle inoculation | 2 |
