# Supplementary material for: Characterising Foot-and-Mouth Disease Virus in Clinical Samples Using Nanopore Sequencing
Source: Front Vet Sci. 2021 May 17;8:656256. doi: 10.3389/fvets.2021.656256 (PMC8165188; doi:10.3389/fvets.2021.656256)
Supplement: Supplementary file 3 [file Table_3.DOCX]

**Table S.3:** The total yield of DNA per sample as determined by the Qubit v3 fluorometer.

| Sample ID | Total yield (ng) | Sequencing run |
| --- | --- | --- |
| O/UKG-CC | 1296 | Run 1 |
| O/UKG-OS-1 | 761 | Run 2 |
| O/UKG-OS-2 | 163 | - |
| O/UKG-OS-3 | 5 | - |
| O/UKG-OS-4 | 286 | - |
| O/UKG-EPI-1 | 1281 | Run 2 |
| O/UKG-EPI-2 | 613 | - |
| A/TAI-CC | 1204 | Run 1 |
| A/TAI-OS-1 | 299 | - |
| A/TAI-OS-2 | 902 | Run 2 |
| A/TAI-OS-3 | 977 | - |
| A/TAI-OS-4 | 824 | - |
| A/TAI-EPI-1 | 1151 | - |
| A/TAI-EPI-2 | 1125 | Run 2 |
| ASIA1/IRN-CC | 1204 | Run 1 |
| ASIA1/IRN-OS-1 | 332 | - |
| ASIA1/IRN-OS-2 | 400 | Run 2 |
| ASIA1/IRN-OS-3 | 629 | - |
| ASIA1/IRN-OS-4 | 442 | - |
| ASIA1/IRN-EPI-1 | 1138 | Run 2 |
