# Supplementary material for: Characterising Foot-and-Mouth Disease Virus in Clinical Samples Using Nanopore Sequencing
Source: Front Vet Sci. 2021 May 17;8:656256. doi: 10.3389/fvets.2021.656256 (PMC8165188; doi:10.3389/fvets.2021.656256)
Supplement: Supplementary file 4 [file Data_Sheet_1.PDF]

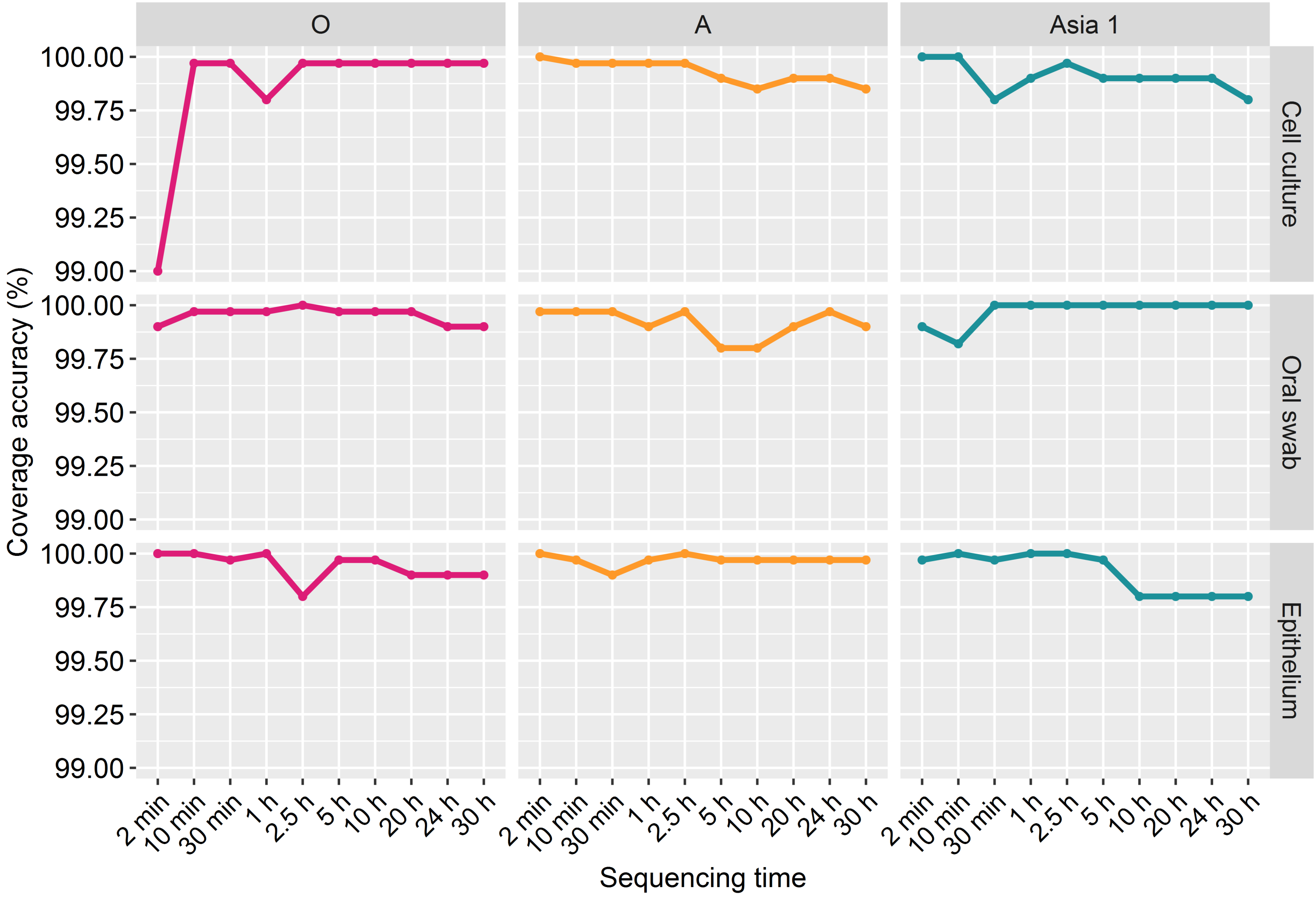

Figure S.1: Consensus accuracy (%) achieved for each sample throughout the duration of the MinION sequencing runs when compared to the respective reference sequence generated on the Illumina MiSeq.
